# Water-soluble microencapsulation using gum Arabic and skim milk enhances viability and efficacy of *Pediococcus acidilactici* probiotic strains for application in broiler chickens

**DOI:** 10.5713/ab.23.0446

**Published:** 2024-04-01

**Authors:** Ratchnida Kamwa, Benjamas Khurajog, Nongnuj Muangsin, Pawiya Pupa, David J Hampson, Nuvee Prapasarakul

**Affiliations:** 1 Department of Microbiology, Faculty of Veterinary Science, Chulalongkorn University, Bangkok 10330, Thailand; 2 The International Graduate Course of Veterinary Science and Technology, Faculty of Veterinary Science, Chulalongkorn University, Bangkok 10330, Thailand; 3 Department of Chemistry, Faculty of Science, Chulalongkorn University, Bangkok 10330, Thailand; 4 Department of Biology, Faculty of Science, Chulalongkorn University, Bangkok 10330, Thailand; 5 School of Veterinary Medicine, Murdoch University, Perth, Western Australia, 6150, Australia; 6 Center of Excellence in Diagnosis and Monitoring Animal Pathogens (DMAP), Faculty of Veterinary Science, Chulalongkorn University, Bangkok 10330, Thailand

**Keywords:** Antibiotic-alternative, Broiler Chicken, Microencapsulation, Probiotics, *Salmonella*, Spray Dry

## Abstract

**Objective:**

This study aimed to develop and evaluate the effectiveness of a water-soluble microencapsulation method for probiotic strains using gum Arabic (GA) and skim milk (SKM) over a three-month storage period following processing.

**Methods:**

Four strains of *Pediococcus acidilactici* (BYF26, BYF20, BF9, and BF14) that were typical lactic acid bacteria (LAB) isolated from the chicken gut were mixed with different ratios of GA and SKM as coating agents before spray drying at an inlet temperature 140°C. After processing, the survivability and probiotic qualities of the strains were assessed from two weeks to three months of storage at varied temperatures, and de-encapsulation was performed to confirm the soluble properties. Finally, the antibacterial activity of the probiotics was assessed under simulated gastrointestinal conditions.

**Results:**

As shown by scanning electron microscopy, spray-drying produced a spherical, white-yellow powder. The encapsulation efficacy (percent) was greatest for a coating containing a combination of 30% gum Arabic: 30% skim milk (w/v) (GA:SKM30) compared to lower concentrations of the two ingredients (p<0.05). Coating with GA:SKM30 (w/v) significantly enhanced (p<0.05) BYF26 survival under simulated gastrointestinal conditions (pH 2.5 to 3) and maintained higher survival rates compared to non-encapsulated cells under an artificial intestinal juices condition of pH 6. De-encapsulation tests indicated that the encapsulated powder dissolved in water while keeping viable cell counts within the effective range of 10^6^ for 6 hours. In addition, following three months storage at 4°C, microencapsulation of BYF26 in GA:SKM30 maintained both the number of viable cells (p<0.05) and the preparation’s antibacterial efficacy against pathogenic bacteria, specifically strains of *Salmonella*.

**Conclusion:**

Our prototype water-soluble probiotic microencapsulation GA:SKM30 effectively maintains LAB characteristics and survival rates, demonstrating its potential for use in preserving probiotic strains that can be used in chickens and potentially in other livestock.

## INTRODUCTION

Animal production is increasingly challenged by disease threats, and this is exemplified in the poultry industry which is grappling with the problem of ensuring the health and safety of broiler chickens, especially in the face of threats from enteric pathogens such as *Salmonella*. Not only does *Salmonella* jeopardize animal health, production and welfare, but it also poses significant risks to public health as it is a formidable zoonotic foodborne pathogen [1]. Alarmingly, it has become more difficult to control *Salmonella* due to an increase in antibiotic-resistant strains.

The use of probiotics has emerged as a promising tool in addressing the challenge of controlling infections with resistant enteric pathogenic bacteria. Probiotics are live microorganisms that can be delivered orally to confer health benefits to their host, ranging from enhancement of the immune response to pathogen inhibition [2]. Dietary supplementation with lactic acid bacteria (LAB), a subset of probiotics, has shown potential in mitigating *Salmonella* infections by promoting beneficial bacterial populations in the gastrointestinal tract and by producing antimicrobial compounds [3,4]. In addition, supplementing the feed of meat chickens (broilers) with the probiotic *Lactobacillus acidophilus* improved the feed conversion ratio and body weight gain compared to birds not receiving the supplement [5].

Identifying bacterial strains that are appropriate for use as probiotics in different animal species and health conditions is only part of the challenge in developing this means of assisting disease control. Delivering such probiotics effectively remains a hurdle. One method to help achieve this is encapsulation, a method that envelops probiotics in small capsules to protect them from adverse conditions [6]. Probiotic encapsulation technologies encompass physical methods such as spray drying, freeze-drying, spray chilling, spray cooling, extrusion, fluidized bed drying, electrospraying, and electrospinning, as well as chemical methods like coacervation, ionic gelation, and molecular inclusion [7]. Of these techniques, spray drying stands out due to its scalability, cost-effectiveness, and ability to extend the shelf life of probiotics [8]. This method involves atomizing a probiotic suspension into a stream of heated air, resulting in protective spherical microcapsules. Wall materials, such as gum Arabic (GA: a gum from the acacia tree) and whey protein in skimmed milk (SKM), play an essential role in maintaining the integrity of these capsules during the process [9].

Probiotic encapsulation efficiency (EE) serves as a crucial parameter to assess the effectiveness of the encapsulation process, which depends on factors such as particle size, wall materials, and the chosen encapsulation technique. The selected wall materials should be non-toxic, food-grade, able to provide protection against environmental factors, and capable of controlling the release of encapsulated probiotics [10]. Several materials that are used for encapsulation are certified as “generally recognized as safe” (GRAS), and they include proteins (casein, gelatin, skim milk etc.) and polymers (gum Arabic, alginate, cellulose, etc.) [11]. Water-soluble microcapsules in particular promise targeted release and increased efficacy as they dissolve predictably in the digestive tract, ensuring a consistent supply of beneficial microorganisms [12]. This predictability translates to improved health and growth performance outcomes when used in young animals such as broiler chickens [13]. Given the promise of probiotics in combatting *Salmonella* infections and the potential of spray drying, the aim of our study was to develop water-soluble micro-encapsulations using GA and SKM and test these coatings with probiotic strains. We assessed the probiotics properties of the encapsulated strains, from shelf life to antibacterial activity against *Salmonella*, envisioning a future where this technology can be used to enhance probiotic survival, provide long-term stability, offer water solubility and bioavailability, contribute to disease control, and potentially extend its application beyond poultry farming. These aspects collectively make it a promising innovation for improving the health and performance of broiler chickens in their crucial early stage of production, and potentially has application in other livestock species.

## MATERIALS AND METHODS

### Experimental ethics

The experimental protocol was approved by the Institutional Biosafety Committee of the Faculty of Veterinary Science, Chulalongkorn University (Protocol Review No. IBC-2331048).

### Probiotic strain cultivation

*Pediococcus acidilactici* strains BYF26, BYF20, BF14, and BF19 were originally isolated from broiler feces in Thailand [14]. These strains have been shown to have multiple potential probiotic functions, including resistance to acid and bile, absence of antimicrobial-resistance genes based on criteria established by the European Food Safety Authority (EFSA), and demonstrated antibacterial properties against *Salmonella enterica* [14]. The probiotic strains were incubated anaerobically in De Man Rogosa and Sharpe (MRS) broth (Difco, Sparks, MD, USA) for 48 h at 37°C [15]. After incubation, cells were harvested by centrifuging at 2,000 rpm for 10 minutes at 4°C and washing in 0.1% peptone water (Difco, USA). The LAB cells were stored at −20°C in MRS broth supplemented with 20% (w/v) glycerol until used.

### Media preparation for drying

Gum arabic (GA; BKK chemical, Bangkok, Thailand) and skim milk powder (SKM; Dairy Rich, Samut Prakarn, Thailand) were utilized as microencapsulation wall materials. Table 1 lists the formulation used for microencapsulation of *P. acidilactici* BYF26. GA and SKM were dispersed individually in distilled water at concentrations of 10, 20, and 30 g weight by volume, and both wall materials were blended in a homogenizer. Formulations included mixtures of 10%, 20%, and 30% of both components, as well as 30% GA and 30% SKM used alone (i.e. five formulations in total). The wall material solutions were heated to 80°C for 30 minutes before being cooled to ambient temperature [16]. A fresh BYF26 cell culture with more than 9 log colony-forming units (CFU/mL) was mixed with the wall material formulations at a ratio of 1:5 (v/v) prior to processing, and the mixture was then homogenized [17]. The optimal concentration of wall material was selected and used to prepare the other probiotic strains BYF20, BF9, and BF14 for comparison in all subsequent experiments.

### Spray drying

The spray drying process was carried out utilizing a laboratory-scale spray dryer (Mini spray dryer B-290; Buchi, Flawil, Switzerland) with an inlet temperature of 140°C and an outlet temperature of around 80°C to 90°C [18]. The probiotic powder was collected in sterile corning tubes (NEST, Wuxi, China) and stored at 4°C and at room temperature (approximately 25°C to 35°C in Thailand) for testing in subsequent experiments.

### Enumeration of microencapsulated *P. acidilactici* (BYF26, BYF20, BF9, and BF14) and encapsulation efficacy

To determine the viability of the microencapsulation powder following the process, the probiotics were released from the microcapsules using a modified version of the method described by Zaeim et al [19]. One milliliter of the combined solution prior to spray drying and 0.1 g of the encapsulating powder were resuspended in 10 mL of 0.1% peptone water and serially diluted using the drop plate method. After 48 h of aerobic incubation at 37°C, the viable cell count was calculated, and viable cells were expressed as log CFU/g.

The encapsulation efficacy was calculated using the equation:


$$\text{EE}\%=({\text{log}}_{10}\;\text{N}/{\text{log}}_{10}\;\text{No})\times 100$$

Where N represents the total number of bacteria in the encapsulation powder and No represents the total number of bacteria in the combined solution prior to spray drying.

Bacterial count experiments were conducted three times, and the results were averaged and reported as the mean and standard deviation. Although there is no standard for capsulation efficiency, it is recommended that probiotic bacteria be used in the range of 10^8^ to 10^9^ CFU/g for consumption by animals and at least 10^6^ CFU/g of viable probiotic cells should be available throughout the product shelf life [20].

### Ultrastructural morphology and size of probiotic microcapsule

A scanning electron microscope (SU3500; Hitadchi Horiba X-maxn, Tokyo, Japan) was used to determine the morphology and size of microencapsulated probiotics, using the protocols described by Fritzen-Freire et al [21]. The microencapsules were adhered to metal stubs using double-sided adhesive tape before being coated with gold using a vacuum sputter coater. Samples were examined at 10 and 15 kV acceleration voltages and a 5,000× magnification.

### De-encapsulation of microencapsulated probiotic BYF26

The method used was modified from a previous study [22]. One gram of microencapsulation powder containing 30 GA:30 SKM BYF26 was resuspended in 10 mL of distilled water and stored at room temperature for six hours as a representative sample. A tenfold dilution in 0.5% peptone water was used in the drop plate method to count viable cells, with samples collected every hour. The experiment was carried out in triplicate.

The survival cells were calculated using the equation:


Surviving cells=log (N/No)

No and N represent the number of viable CFUs/mL at the beginning and conclusion of the collection period, respectively.

### Survival of microencapsulated *P. acidilactici* (BYF26, BYF20, BF9, and BF14) at up to 90-days storage

The survival of the probiotic strains was measured after 14, 30, 60, and 90 days of storage. Samples of probiotic powder (0.1 g) stored at room temperature and 4°C were serially diluted in 10 mL of a 0.1% peptone water solution. Viable cells were counted after dropping suspensions onto MRS agar and incubating at 37°C for 48 hours.

The survival rate was calculated as:


$$\text{Log reduction}=\text{log }{\text{N}}_{\text{b}}-\text{log }{\text{N}}_{\text{a}}$$

Where N_b_ represents the number of viable cells (CFU/g) in the feed solutions before spray drying and N_a_ represents the number of viable cells (CFU/g) in the encapsulation powder after spray drying [22].

### Confirmation of probiotic properties post-encapsulation

#### Survival of microencapsulated P. acidilactici (BYF26, BYF20, BF9, and BF14) under in vitro gastrointestinal conditions

The viability and stability of the probiotic formulations under conditions that simulated the physiological pH in different parts of the broiler gastrointestinal tract (GIT) were investigated. These included artificial gastric juices (AGJ) to simulate conditions in the crop, stomach and gizzard (pH 2.5 to 4.5) and artificial intestinal juices (AIJ) to simulate conditions in the small intestine (pH 6.5) [23,24].

#### Preparation of artificial gastric juices (AGJ) and artificial intestinal juices (AIJ)

The AGJ and AIJ were prepared using previously described methods, with minor modifications [15,25]. The AGJ was produced by dissolving 3 mg/mL of pepsin in 0.85% NaCl sterile saline. The pH of the AGJ was adjusted to 4.5 with 1 M HCl to replicate crop and glandular stomach juice, and to 2.5 to simulate gizzard juice. To create the AIJ, 0.2% NaCl was combined with 1 mg/mL pancreatin and 0.45% bile salt. The pH of the AIJ was adjusted to 6.5 using 0.1 M NaOH. The solution was preserved at 4°C for future use.

#### Release of probiotics from encapsulation under simulated in vivo conditions

The cell viability of the LAB in the supernatant released from the powder that was sequentially exposed to AGJ and AIJ was determined [15]. One gram of probiotic encapsulation powder was mixed with AGJ at a ratio of 1:3 (w/v). The pH was adjusted to 4.5 and the mixture was shaken at 40 rpm in an incubator for two hours at 41°C. The pH of the AGJ was adjusted to 2.5 using HCl, and it was incubated for a further 30 minutes in a shaking incubator. After 2.5 hours of incubation, the AGJ were collected by centrifugation (8,500×g, 15 min, 4°C) and resuspended with the AIJ at a ratio of 1:3 (w/v). The pH of the mixture was adjusted to 6.5 using 0.1 M NaOH, and then it was agitated at 40 rpm for two hours at 41°C. The drop plate method of ten-fold dilution on MRS agar plates was used to determine the viable LAB released into the supernatant of the AGJ and AIJ every 30 minutes. LAB-free cells were utilized in the assay to compare cell viability during successive exposure to AGJ and AIJ with removal of the cells from encapsulation. The experiments were carried out in triplicate. The number of LAB colonies was determined and converted to a percentage of the LAB content in the original sample using the following equation [26]:


Cell release (%)=(N/No)×100

Where No and N represent the number of viable CFU/mL at the initial bacterial count and at the count following release at the different times, respectively.

#### Control strains and their cultivation

Control bacterial strains *Escherichia coli* ATCC 25922, *Staphylococcus aureus* ATCC 25922, and *Salmonella* Typhimurium ATCC 13311 were obtained from the American Type Culture Collection (ATCC; Manassas, VA, USA). Nine serovars of *Salmonella enterica* comprising Typhimurium, Agona, Kentucky, Virchow, Albany, Braenderup, Hadar, Enteritidis and Give were selected as indicator pathogenic strains obtained from chickens on broiler chicken farms in Thailand [14]. All pathogenic strains were cultivated on tryptic soy agar (TSA) at 37°C for 18 h following MALDI-TOF MS confirmation of their identity. Each pathogen then was grown on Mueller Hinton agar (MHA) and a single colony was selected, resuspended in 0.85% NaCl, quantified at 0.5 McFarland standard (about 1.5×10^8^ CFU/mL), and subsequently cultured on MHA.

#### Evaluation of antibacterial activity of microencapsulated probiotics

One gram of freshly encapsulated probiotic powders and the same materials after three-months of storage at 4°C were weighed and decapsulated with peptone water for preparation of cell-free supernatants (CFS). In addition, CFS from the non-encapsulated probiotic strains and the encapsulation material solution (gum Arabic and skim milk mix at a concentration of 30:30 w/v) were used as controls. The antibacterial activity of the CFSs were evaluated using a modified version of the agar well diffusion experiment described by Ayala et al [27]. Before testing, the pH value was measured, and cell free supernatants were obtained by passing through a 0.22 mm syringe filter. After 30 minutes, four 6-mm-diameter wells were punched into each plate containing a lawn of pathogenic bacterial strains (concentration 8 log CFU/mL adjusted by 0.5 MacFarland standard) and the agar was allowed to sit for 10 minutes. Subsequently, 100 mL of the corresponding CFS was added to each well. All plates were incubated at 37°C for 24 h. After incubation, the inhibition zones were measured in millimeters (mm) and categorized as shown in Supplementary Table S1 [28].

### Statistical analysis

All statistical analyses were performed using SPSS version 28 (IBM, New York, USA) and the findings were expressed as the mean, standard deviation, and standard error of the mean (SEM). All data were derived from three independent experiments. The formulation of the encapsulation mean served as the experimental unit, according to the following model:


$${\text{Y}}_{\text{ij}}=\mu +{\text{T}}_{\text{i}}+{\text{e}}_{\text{ij}}$$

Where Y_ij_ is the dependent variable observation, μ is overall mean, T_i_ is the effect of the formulation, and e_ij_ is the random error. All the data were analyzed in the normality test by using the Shapiro-Wilk test and Homogeneity of variance test to confirm the data homogeneity. The quantity of viable bacterial cells, the EE percentage, and the Log of viability reduction were analyzed using a one-way analysis of variance (ANOVA) for the homogeneity data and a one-way ANOVA with Dunnett T3 for the non-homogeneity data. Tukey’s Post Hoc tests were utilized for comparison after ANOVA testing. The statistical significance threshold was established at p<0.05.

## RESULTS

### Microencapsulation efficacy

The encapsulation efficacies ranged from 73% to 90% for the LAB strain BYF26 across various material concentrations (as detailed in Table 2). Notably, dual-material microencapsulation, employing a combination of 30% GA and 30% SKM (GA:SKM30), displayed the highest efficacy at approximately 90%. This was significantly higher (p<0.05) than for formulations using single materials. Differences between the encapsulation efficacies for the four *P. acidilactici* strains were not statistically significant (Supplementary Table S2).

### Morphology and size of microcapsules

**Figure 1:**
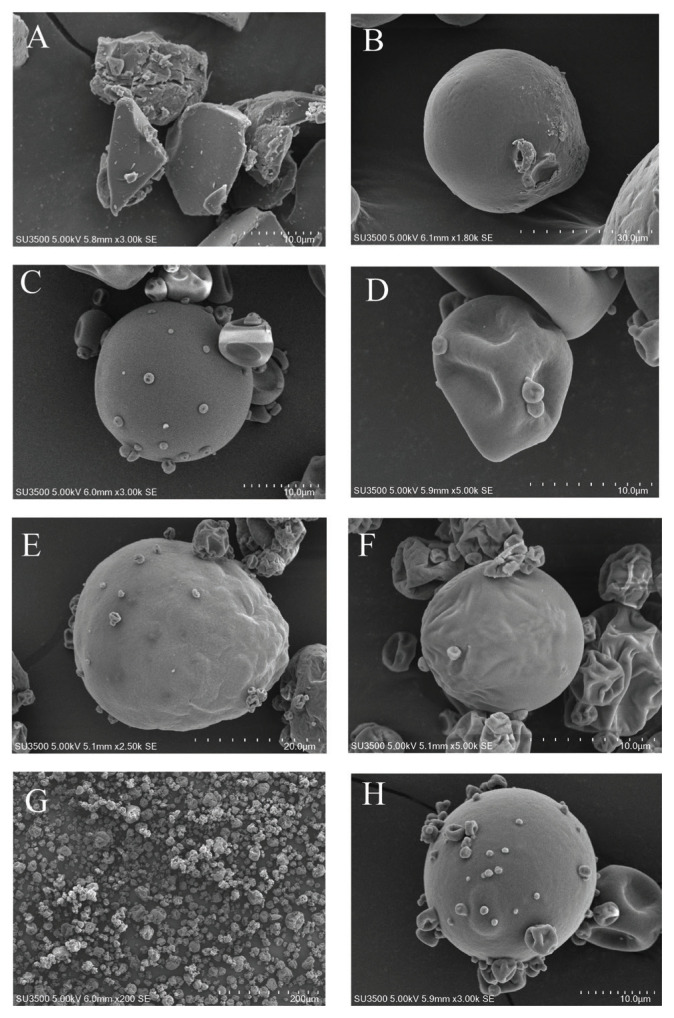
Scanning electron microscope images of BYF26 with different formulations and comparing the material before and after encapsulation. Before encapsulation (A) Gum Arabic and (B) Skim milk powder. After the encapsulation process (C) GA:30, (D) SKM:30, (E) GA:SKM 10, (F) GA:SKM20, (G) overview of GA:SKM30, and (H) GA:SKM30, respectively.

Standard error of the mean imaging revealed that all encapsulation formulations yielded predominantly spherical powdery particles with varying magnifications (Figure 1B-H). The unencapsulated GA material (Figure 1A) exhibited a crystalline morphology, contrasting starkly with the spherical shape observed post-encapsulation (Figure 1C). On the other hand, the morphology of the skim milk remained consistent pre- and post-encapsulation (Figure 1B and D). A broad overview of the encapsulated powder revealed particles varying in size (Figure 1G), but all particles were in the micrometer size range, and all were smaller than 50 μm in all encapsulation formulations.

### De-encapsulation

**Figure 2:**
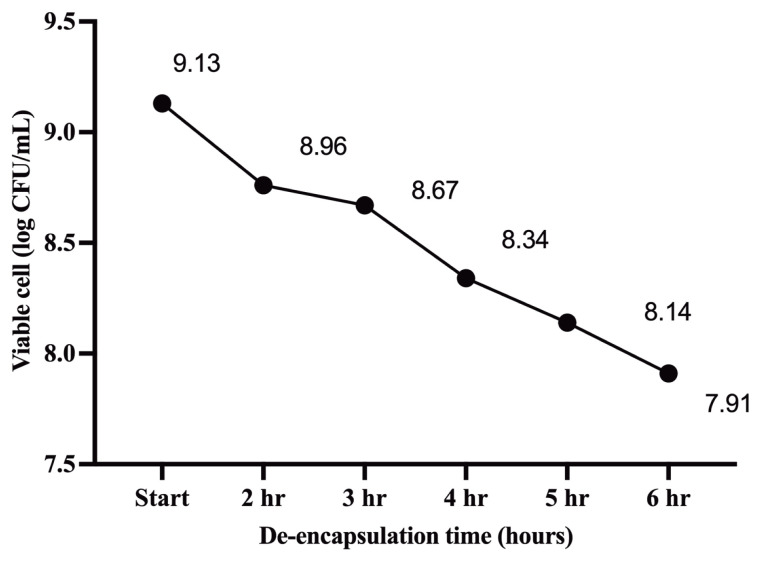
Cell survival of probiotic formulation GA:SKM30 BYF26 after de-encapsulation for six hours at room temperature. The data plot utilizes the mean of 3 replicates in log CFU/mL units. GA:SKM30, 30% gum Arabic: 30% skim milk (w/v).

Assessing post de-encapsulation of the BYF26 encapsulation formula GA:SKM30 in water over a duration of six hours revealed a decline in viable cell counts by approximately 2 log CFU/mL (Figure 2). Nevertheless, the post de-encapsulation samples maintained a high live cell probiotic count, exceeding 6 log CFU/mL.

### Viability test

Results for the viability of the four *P. acidilactici* strains encapsulated with varying concentrations of GA and SKM assessed over a 90-day period at both room temperature and 4°C are shown in Table 3 and Supplementary Table S3. The viability of both the single-material encapsulations was significantly less (p<0.05) than for all the dual-material encapsulations at both storage temperatures. Formulations 4 (GA:SKM20) and 5 (GA:SKM30) exhibited the highest post-encapsulation viability at both storage temperatures. For these formulations, there was a decline of around 1–2 log CFU/mL after 60 days of storage, yet the cell counts remained robust, exceeding 6 log CFU/mL. In contrast, unencapsulated LAB cells became non-viable after 7 days of storage at room temperature and one month at 4°C.

Further viability analysis across different LAB strains identified significant differences (p<0.05) between storage temperatures (4°C and room temperature) by the 60th day, with storage at 4°C promoting viability. By 90 days none of the strains in any of the formulations stored at room temperature had survived. Overall, counts of strain BYF26 remained consistent across both storage conditions. Encouragingly, after 90 days of storage at 4°C all LAB strains encapsulated with GA:SKM30 maintained cell counts exceeding 6 log CFU/mL.

### Confirmation of probiotic properties post-encapsulation

#### Performance in simulated gastrointestinal (GIT) conditions

**Figure 3:**
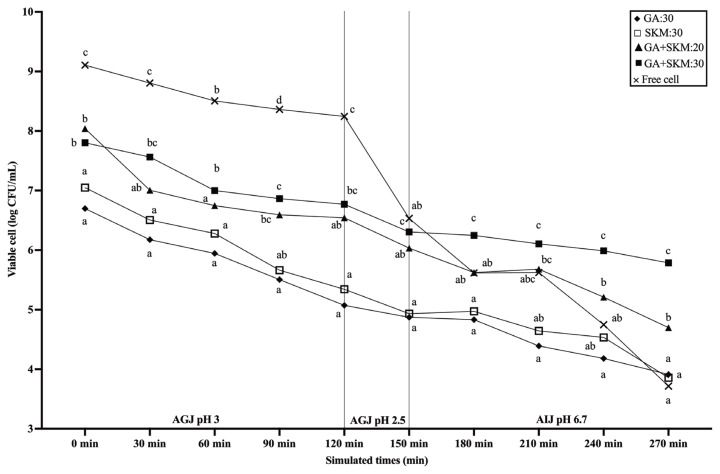
Comparison of the cell survival of different formulations of encapsulated probiotic strains and free cells incubated under simulated gastrointestinal tract (GIT) conditions. Data points are the means of three replicates. For each time point, counts marked with different lowercase letters are statistically significantly different (p<0.05).

After a 90-day storage period at 4°C, the encapsulation efficacy of single material formulations (either GA or SKM) were compared against those for double material formulations (combinations of GA:SKM at concentrations of 20% and 30%). This was tested under simulated gastrointestinal conditions with AGJ at pH 3 and pH 2.5, and AIJ at pH 6.7. The viability varied across formulations, especially at the 270-minute mark in the AIJ (Figure 3). Notably, the double material encapsulations demonstrated a significant advantage (p<0.05) in viability over both the free cells and single material encapsulations. While free cells maintained the highest viability during the pH 3 AGJ stage, they exhibited a decline of nearly 2 log CFU/mL upon transitioning to a pH of 2.5. This reduction was steeper compared to those observed with GA:SKM formulations, yet all retained viability counts exceeding 6 log CFU/mL. After 270 minutes, the GA:SKM30 formulation stood out, showing the highest viability count at approximately 6 log CFU/mL. Furthermore, when analyzing the viability across different LAB strains, BYF26 and BYF20 demonstrated significantly greater (p<0.05) counts compared to the other two LAB strains (Supplementary Figure S1).

#### Evaluation of antibacterial activity of microencapsulated probiotics

The antibacterial efficacies of the CFSs from the four *P. acidilactici* strains before encapsulation, after encapsulation in the GA:SKM30 formulation, and after encapsulation and storage for 90 days at 4°C are summarized in Table 4. The CFSs exhibited antibacterial action against the ATCC and *Salmonella* strains, ranging from moderate to strong inhibition, even after lengthy storage. *P. acidilactici* strains BY9 and BF14 only modestly inhibited the growth of certain of the *Salmonella* serovars (i.e., Give, Virchow, and Agona), whereas the other two strains showed stronger activity against most of the *Salmonella* strains. The antibacterial activity of the *P. acidilactici* strains tended to be slightly lower after encapsulation, but the activity was largely retained after 3 months of storage. The control encapsulate solution (gum Arabic mix with skim milk at ratio 30:30 w/v) did not inhibit the indicator pathogenic strains.

## DISCUSSION

The effectiveness of probiotic microencapsulation in animal supplements hinges on multiple factors: the probiotic’s viability during its shelf-life, its resilience against harsh gastrointestinal conditions, and its overall properties that promote gut health. Our study sought to develop a water-soluble encapsulation prototype of probiotic *P. acidilactici* using varying concentrations of GA and SKM. The intention was to identify an optimal formulation that would ensure probiotic survival post-encapsulation and extend the shelf life of the product. Hence, we assessed the prototype’s potential to enhance probiotic viability during a 90-day storage period and its performance in a simulated chicken intestinal environment.

Our encapsulation efficacy results using GA and SKM in the formulation GA:SKM30 gave the highest EE percentage at 90%, but this was somewhat lower than the results reported by Reddy et al [29], where an efficacy of over 97% was achieved using reconstituted skim milk and maltose dextrin as wall material. In their study the outlet temperature was around 40°C, while in our study the outlet temperature was around 80°C to 90°C. The use of a lower outlet temperature might increase the viable cell count of probiotic cells and increase the encapsulation efficacy, and this requires further investigation. The double wall material coating in our study gave an encapsulation efficacy of around 90%, which was greater than with a single wall material coat, and which aligned more closely with findings by Rajam and Anandharamakrishnan who showed efficacy percentages ranging from 70.77% to 72.82% using only fructo-oligosaccharide as a wall material [8]. While our findings present a slightly lower encapsulation efficacy than can be achieved, it is essential to consider the broader implications. The GA and SKM utilized in our study previously have been highlighted for their potential to enhance cell survival during high inlet and outlet temperatures in the spray drying process. Substances in skim milk such as whey protein may provide a protective coating on the bacterial cell wall during the spray drying process [30,31]. This suggests that our chosen materials and methods may offer advantages as a prebiotic component that benefits probiotic bacteria both under GIT conditions and in specific processing conditions or applications.

In the spray drying process, probiotic suspensions were atomized into a flow of heated air in the spray drier chamber. The GA and SKM solution acted as the protective coating against thermal denaturation during the encapsulation process, covering the probiotic cells to produce a probiotic powder [6,10]. In every instance, SEM examination demonstrated the effective development of microencapsulation for powdered GA and SKM. Spherical particles were detected with an average size the same as that obtained by Fang and Bhandari [32], who determined that the average size of spray-dried powder is between 10 and 100 μm, and that the concentration of the wall material can confirm the particle size. For the morphology, they hypothesize that rapid atomizing of the probiotic solution’s water during the spray-drying procedure likely contributes to the shape of the encapsulating powder. As shown by Rodríguez-Restrepo et al [33], the morphology of microcapsules in general does not exhibit characteristics that are specific to the kind of wall material.

This study generated a water-soluble probiotic capsule for use in poultry by combining GA and SKM. GA is renowned for its water-solubility and stabilizing capabilities, and it forms stable colloidal solutions upon water dispersion. Its viscosity, conferred by its ability to hydrate and swell, makes GA a versatile candidate for emulsification, encapsulation, and thickening applications [25]. Conversely, SKM, due to its soluble protein and lactose content, dissolves seamlessly in water, creating a homogenous solution. The protein, primarily casein, not only supports the water-solubility of skim milk but also ensures solution stabilization [34]. In the arena of probiotic encapsulation tailored for poultry and other livestock, these materials can craft protective matrices around probiotics, ensuring they remain stable and viable during administration.

Storage conditions play a pivotal role in determining the longevity and functionality of encapsulated probiotics. Environmental stressors, like heat, moisture, and oxidation, can compromise probiotic physiology and functionality [35]. Our results emphasize the superiority of storage at 4°C for preserving probiotic properties. In contrast, room temperature storage (approximately 25°C to 35°C in Thailand) jeopardizes probiotic viability, likely contributed to by moisture uptake which can result in oxidation of membrane lipids [36]. Probiotic bacteria are often anaerobic or facultative anaerobic, and exposure to oxygen during storage can lead to oxidative stress and reduce viability [37]. Oliveira et al [38] founded that encapsulated *Bifidobacterium lactis* showed a decrease in viable cell count of 6 log CFU/mL after storage for 30 day at room temperature (35°C to 37°C), and recommend storage at 7°C for probiotic microencapsulation. Extending this observation, we infer that GA and SKM encapsulation potentially prolongs probiotic shelf life beyond six months when stored at 4°C, substantiating research from Broeckx et al [39]. Importantly, the viability of different *P. acidilactici* strains remained consistent throughout the storage period at 4°C.

For probiotics to confer their health benefits, they must traverse the harsh conditions of the GIT and reach the small intestine in substantial numbers. Mimicking the acidic environment of the chicken GIT, our simulated gastric solution presented a challenging pH range of 2.5 to 4. In this setup, encapsulated probiotics showed considerable resilience, maintaining a viable cell count exceeding 6 log 10 CFU/mL. Notably, encapsulated cells consistently outperformed their free counterparts, echoing findings from Leylak et al [40]. It is widely believed that the longer a probiotic strain remains in the GIT, the more chances it has to exert beneficial effects. The ability of a strain to adhere to intestinal mucus is considered to be linked to its intestinal residence time, and is a prerequisite for temporal colonization of the mucosal surface according to Servin et al [41]. Moreover, our selected strains of *P. acidilactici* demonstrated high survival rates under low pH conditions (2.5 for 3 h) and high bile salt concentration (0.7% for 6 h). Additionally, they showed tolerance to phenol, which is produced by commensal bacteria in the GIT and which can inhibit LAB growth as described by Khurajog et al [14].

Assessing antibacterial activity post-encapsulation revealed a slight reduction in efficacy post-processing. This variance, contingent on both probiotic strain and target pathogen, aligns with the findings of Pupa et al [17] suggesting that active microbial compounds may remain confined within the encapsulated matrix, reducing their availability in the CFS. However, *P. acidilactici* demonstrated pronounced antibacterial activity against *S*. Typhimurium, presumably by producing anti-microbial substances such as hydrogen peroxide, organic acids, and bacteriocin, as described by Seo and Kang [42]. Moreover, bacterial species such as *E. coli* and *Salmonella* are not tolerant of acidic environments, thus resulting in increased susceptibility to the actions of bacteriocins and short chain fatty acid produced by probiotic strains [43]. These findings highlight the potential of *P. acidilactici* as a biocontrol agent. Thus, our encapsulation approach, optimized with suitable wall materials, showcases promising antibacterial preservation, underscoring its potential for industrial applications in poultry, and potentially in other livestock species.

## CONCLUSION

We created a model for delivering probiotics to chickens by utilizing the features of gum Arabic and skim milk. Our findings demonstrate the importance of selecting optimal encapsulating materials and storage conditions to ensure the efficacy and lifespan of encapsulated probiotics. Our optimal formulation was GA:SKM30 used with probiotic strains *P. acidilactici* BYF26 or BYF20, and this gave a high viable cell count of more than 6 log CFU/mL after 90 day storage at 4°C. De-encapsulation confirmed the soluble properties of the encapsulated material, with cell counts of 6 log CFU/mL after 6 hours. The encapsulated probiotics not only demonstrated resilience in simulated gastrointestinal conditions but also showed pronounced antibacterial activity, particularly in inhibiting *Salmonella* strains. Future efforts in this field should concentrate on perfecting the encapsulating method to match the requirements of different chicken ages and feed types, broadening the spectrum of probiotics examined, and conducting *in vivo* trials to achieve the full potential of this strategy.

## Figures and Tables

**Table 1:** Formulation used for media preparation of coating material on a weight by volume basis

Formula	Material ratio	Gum Arabic % (w/v)	Skim milk % (w/v)
1	GA30	30	-
2	SKM30	-	30
3	GA:SKM10	10	10
4	GA:SKM20	20	20
5	GA:SKM30	30	30

GA, gum Arabic; SKM, skim milk; w/v; weight by volume.

**Table 2:** Encapsulation efficacy for *P. acidilactici* strain BYF26 with different formulations of wall material^1)^

Formula	Material ratio	Encapsulation efficacy (%)
1	GA30	73.68±3.28^a^
2	SKM30	73.88±0.97^a^
3	GA:SKM10	84.27±0.93^b^
4	GA:SKM20	86.49±1.43^bc^
5	GA:SKM30	90.13±0.77^c^
SEM		1.84
p-value		<0.001

SEM, standard error of mean; GA, gum Arabic; SKM, skim milk.

1) Encapsulation efficacy data represent the mean of 3 replicates±standard deviation.

a–c Different lowercase letters within each column indicate a significant difference between formulations (p<0.05).

**Table 3:** Survival of *P. acidilactici* BYF26 with single and double wall materials during a 90-day storage period^1)2)^

Time (d)	GA:30	SKM:30	GA:SKM10	GA:SKM20	GA:SKM30	SEM	p-value
0	7.89±0.36^a^	8.80±0.08^a^	9.76±0.02^b^	9.88±0.13^bc^	10.82±0.4^c^	1.84	<0.001
Storage at room temperature							
14	7.03±0.06^a^	8.10±0.09^ab^	8.89±0.18^ab^	9.7±0.03^b^	10.04±0.05^ab^	0.23	<0.001
30	6.64±0.57^a^	7.57±0.03^ab^	8.55±0.49^abc^	8.92±0.03^abcd^	9.93±0.11^abcd^	0.23	<0.001
60	6±0.03^bcd^	5.71±0.10^a^	6.67±0.47^ab^	7.37±0.57^bc^	8.45±0.36^cde^	0.25	<0.001
90	Non-viable cells found on the plate					-	-
Storage at 4°C							
14	6.87±0.36^a^	8.30±0.63^ab^	9.66±0.05^b^	9.7±0.10^b^	10.54±0.19^b^	0.23	<0.001
30	6.73±0.04^abcd^	7.7±0.02^abc^	9.41±0.28^d^	9.29±0.02^bcd^	10.29±0.06^cd^	0.23	<0.001
60	6.40±0.52^efg^	6.93±0.16^cde^	8.21±0.10^def^	8.93± 0.22^fg^	10.1±0.01^g^	0.25	<0.001
90	5.4±0.52^a^	5.93±0.16^a^	7.86±0.51^b^	8.61±0.51^b^	9.85±0.22^c^	0.37	<0.001

SEM, standard error of mean; GA, gum Arabic; SKM, skim milk

1) Encapsulation efficacy data represent the mean of 3 replicates±standard deviation in log CFU/mL units.

2) The standard viable cell count of probiotic products after storage time should not be less than 6 log CFU/mL.

a–g Different lowercase letters indicates significant differences at the same storage time (p<0.05).

**Table 4:** Inhibitory effects of cell free supernatant from *P. acidilactici strains* (BYF26, BYF20, BF14, and BF9) against pathogenic bacteria^1)^

Indicator strains^5)^	Before encapsulation^2)^				After encapsulation^3)^				After encapsulation and storage for 90 days^4)^			
		
	BF9	BF14	BYF20	BYF26	BF9	BF14	BYF20	BYF26	BF9	BF14	BYF20	BYF26
*E. coli* ATCC25922	++++	+++	++++	++++	+++	++	++++	+++	+++	++	++++	+++
*S. aureus* ATCC25923	+++	+++	++++	+++	+++	+++	++++	++	+++	+++	++++	++
*S*. Typhimurium ATCC13311	++++	++++	+++	+++	+++	+++	+++	++++	+++	+++	+++	++++
*S*. Typhimurium	++	++	+++	++	+++	++	+++	++	+++	++	+++	++
*S*. Agona	+	+++	++++	+++	+	++	+++	+++	+	++	+++	+++
*S*. Kentucky	++	++	+	++	++	++	+	++	++	++	+	++
*S*. Virchow	+++	++	+++	++++	++	+	++	++	++	+	++	++
*S*. Albany	++	+++	++	++	++	++	++	++	++	++	++	++
*S*. Braenderup	+++	+++	++	+++	++	++	++	+++	++	++	++	+++
*S*. Hadar	+++	+++	+++	+++	++	++	++	+++	++	++	++	+++
*S*. Enteritidis	++	++	+++	++++	++	++	++	++++	++	++	++	++++
*S*. Give	++	++	++	+++	+	++	++	+++	+	++	++	+++

No inhibition zones were found when using the encapsulate solution (gum Arabic mix with skim milk at concentration 30:30 w/v) instead of cell free supernatant

1) Inhibition zone (mm): no inhibition (−); mild (+) (6 to 9); ++, moderate (10 to13); +++, strong (14 to16); ++++, very strong (>17).

2) Original cell free supernatant produced from free cells before encapsulation.

3) Cell free supernatant produced from GA:SKM30 encapsulation formulation after the encapsulation process.

4) Cell free supernatant produced from GA:SKM30 encapsulation formulation after 90 days storage at 4°C.

5) The indicators are pathogenic species and strains that can cause problems in the intestinal tract of poultry. The first three are ATCC strains and the other nine strains of *Salmonella enterica* strains isolated from broiler feces on a farm in Thailand.

## SUPPLEMENTARY MATERIAL

**Supplementary Table S1**. Classification of inhibition zones as referenced by Sirichokchatchawan et al., 2017

**Supplementary Table S2**. Encapsulation efficacy of GA:SKM30 formulations with the four different *P. acidilactici* strains

**Supplementary Table S3**. Survival of four strains of *P. acidilactici* (BYF26, BYF20, BF14, and BF9) encapsulated with GA:SKM30 over a 90 day storage time

**Supplementary Figure S1**. Comparison of the survival of four encapsulated probiotic strains over time under simulated GIT conditions.
