# Supplementary material for: Water-soluble microencapsulation using gum Arabic and skim milk enhances viability and efficacy of Pediococcus acidilactici probiotic strains for application in broiler chickens
Source: Anim Biosci. 2024 Apr 1;37(8):1440–51. doi: 10.5713/ab.23.0446 (PMC11222858; doi:10.5713/ab.23.0446)
Supplement: Supplementary file 1 [file ab-23-0446-Supplementary-Table-1.pdf]

Supplementary Table S1. Classification of inhibition zones, as referenced by Sirichokchatchawan et al., 2017.

**Inhibition zone (mm)**	**Result description**
**6 - 9**	+ : weak inhibition
**10 - 13**	++ : intermediate
**14 - 16**	+++ : strong
**≥ 17**	++++ : extremely strong

mm, millimeter

Supplementary Table S2: Encapsulation efficacy of the GA:SKM30 formulation with four *P. acidilactici* strains.

Encapsulated strains	Encapsulation efficiency (%)
