# Supplementary material for: Water-soluble microencapsulation using gum Arabic and skim milk enhances viability and efficacy of Pediococcus acidilactici probiotic strains for application in broiler chickens
Source: Anim Biosci. 2024 Apr 1;37(8):1440–51. doi: 10.5713/ab.23.0446 (PMC11222858; doi:10.5713/ab.23.0446)
Supplement: Supplementary file 3 [file ab-23-0446-Supplementary-Table-3.pdf]

Supplementary Table S3. Survival of four strains of *P. acidilactici* (BYF26, BYF20, BF14, and BF9) encapsulated with GA:SKM30 over a 90 day storage time[1)2)]

Time (days)	BYF26	BYF20	BF14	BF9	SEM	p-value
**0**	10.82 ± 0.4^{c}	10.62 ± 0.05^{ab}	10.76 ± 0.36^{ab}	11.53 ± 0.59^{ab}	0.14	0.050
**Storage at room temperature**						
**14**	10.04 ± 0.05^{cd}	8.69 ± 0.04^{a}	8.84 ± 0.04^{ab}	9.97 ± 0.57^{cd}	0.15	< 0.001
**30**	9.93 ± 0.11^{cd}	7.98 ± 0.06^{a}	9.97 ± 0.02^{a}	9.65 ± 0.57^{bcd}	0.20	< 0.001
**60**	8.45 ± 0.36^{b}	6.78 ± 0.04^{a}	6.43 ± 0.01^{a}	8.59 ± 0.11^{b}	0.26	< 0.001
**90**		Non-viable cells found on the plate				
**Storage at 4°C**						
**14**	10.54 ± 0.19^{d}	9.59 ± 0.57^{bc}	9.62 ± 0.02^{bc}	10.59 ± 0.13^{d}	0.15	< 0.001
**30**	10.29 ± 0.06^{d}	9.20 ± 0.72^{bc}	8.78 ± 0.07^{ab}	10.42 ± 0.39^{d}	0.20	< 0.001
**60**	10.1 ± 0.01^{d}	9.01 ± 0.06^{bc}	8.33 ± 0.65^{b}	9.81 ± 0.07^{cd}	0.26	< 0.001
**90**	9.85 ± 0.22^{c}	8.65 ± 0.07^{b}	7.93 ± 0.59^{ab}	8.47 ± 0.57^{a}	0.15	0.30

SEM, standard error of mean

[1)] Encapsulation efficacy data represent the mean of 3 replicates ± standard deviation in log CFU/mL units.

[2)] The standard viable cell count of probiotic products after storage time should not be lower than 6 log CFU/mL.

[a-d] Different lowercase letters indicate significant differences at the same storage time ($p < 0.05$).
