# Supplementary material for: Water-soluble microencapsulation using gum Arabic and skim milk enhances viability and efficacy of Pediococcus acidilactici probiotic strains for application in broiler chickens
Source: Anim Biosci. 2024 Apr 1;37(8):1440–51. doi: 10.5713/ab.23.0446 (PMC11222858; doi:10.5713/ab.23.0446)
Supplement: Supplementary file 4 [file ab-23-0446-Supplementary-Fig-1.pdf]

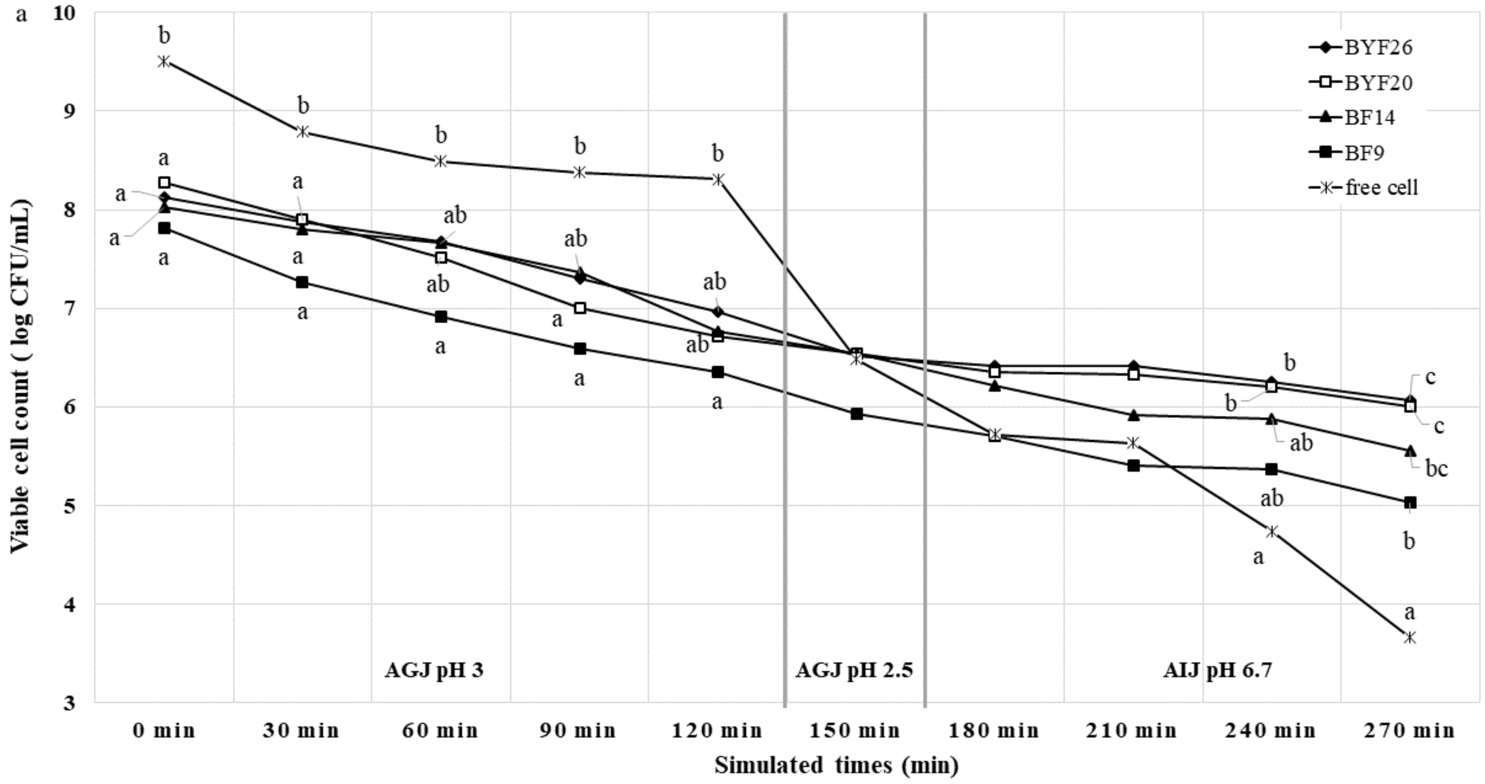

Supplementary Figure S1: Comparison of the survival of four encapsulated probiotic strains over time under simulated GIT conditions. The strains were subjected to three different conditions over a total incubation period of 270 minutes. The experiment is

separated into three parts over time. Strains with different lowercase letters represent statistically significant differences ($p<0.05$) at different times.
